# Back pain was less explained than leg pain: a cross-sectional study using magnetic resonance imaging in low back pain patients with and without radiculopathy

**DOI:** 10.1186/s12891-015-0827-4

**Published:** 2015-12-03

**Authors:** Ole Kudsk Jensen, Claus Vinther Nielsen, Joan Solgaard Sørensen, Kristian Stengaard-Pedersen

**Affiliations:** The Spine Center, Diagnostic Center, Silkeborg Regional Hospital, Falkevej 1-3, 8600 Silkeborg, Denmark; Section of Clinical Social Medicine and Rehabilitation, Institute of Public Health, University of Aarhus, Aarhus, Denmark; Associated to Research Department, Spine Centre of Southern Denmark, Lillebaelt Hospital, Little Belt, Denmark; Department of Rheumatology, Aarhus University Hospital, Aarhus, Denmark

**Keywords:** Low back pain, Leg pain, Magnetic resonance imaging, Disc herniation, Radiculopathy, Nerve root touch, High intensity zone, Osteophytes, Tender points, Widespread pain

## Abstract

**Background:**

Cross-sectional studies have shown associations between lumbar degenerative manifestations on magnetic resonance imaging (MRI) and low back pain (LBP). Disc herniations and other degenerative manifestations, however, frequently occur in asymptomatic individuals. The purpose of this cross-sectional study was to analyze for associations between pain intensity and degenerative manifestations and other pain variables in patients for whom prognostic factors have been published previously.

**Methods:**

Included were 141 consecutive patients with and without radiculopathy, all sick-listed 1–4 months due to low back pain and subsequently examined by MRI of the lumbar spine. Using different methods of grouping the degenerative manifestations, linear regression analyses were performed with the intensity of back + leg pain, back pain and leg pain as dependent variables covering actual pain and pain the preceding 2 weeks. The clinical classification into +/− radiculopathy was established before and independently of the standardised description of MRI findings.

**Results:**

Radiculopathy was present in 43 % of the patients. Pain was best explained using rank-ordered degenerative manifestations on MRI. Back pain and leg pain were differently associated, and back pain was less explained than leg pain in the multivariate analyses (15 % vs. 31 % of the variation). Back pain intensity was higher in patients with type 1 Modic changes and in some patients with nerve root touch, but was not associated with disc herniations. Leg pain intensity was well explained by disc herniations causing MRI nerve root compromise and radiculopathy. In patients with radiculopathy, nerve root touch caused as much leg pain as nerve root displacement or compression. High intensity zones and osteophytes were not associated with back pain, but only associated with leg pain in patients with radiculopathy. Tender points explained some of the back pain, and widespread pain explained leg pain in some of the patients without radiculopathy.

**Conclusions:**

Back pain was associated with type 1 Modic changes, nerve root touch and tender points, whereas leg pain was associated with osteophytes, HIZ, disc herniation, all sorts of MRI nerve root compromise, radiculopathy and widespread pain.

## Background

Cross-sectional population studies have shown statistically significant associations between degenerative manifestations on lumbar magnetic resonance imaging (MRI) and low back pain (LBP) the preceding year [1, 2]. However, degenerative changes occur frequently in persons without LBP [3]. A systematic review including 45 papers found weak associations between LBP and degenerative manifestations with meta-estimates of Odds Ratios ranging from 2.3 to 3.6. [4]. Disc degeneration and disc herniation were identified in 54 % (4–76 %) and 27 % (7–85 %) of persons without LBP, respectively. However, nerve root displacement or compression was only seen in 2–5 % of those without LBP [4], a finding that has been confirmed elsewhere [3]. End plate oedema (Modic changes) was not included in the analyses.

The difficulties in demonstrating clear-cut associations between MRI findings and low back pain may have various explanations: First, MRI cannot differentiate between new and old findings, which may weaken a given association. Second, many studies only look at the presence or absence of degenerative MRI findings which may be inadequate, as more levels of the lumbar spine involved in one person do not count more than one level in another person. A sum score may adjust for this shortcoming [5], but it does not distinguish between persons with many moderate changes and persons with few severe changes, who may have equal sum scores. A rank-ordered classification by the most severely degenerated segment [6] may solve this problem, but then less severely degenerated discs in a person do not count at all.

Finally, back pain may be caused by other mechanisms not necessarily associated with degenerative manifestations. Back pain is part of the definition in chronic widespread pain and fibromyalgia, a subset of this category [7]. The pain in these patients have been shown to be caused by facilitation of pain processing and/or insufficient pain inhibition due to central neuronal mechanisms [8]. We have previously shown that this type of pain mechanism may play a role in a proportion of patients with non-specific LBP [9].

Furthermore, MRI findings have been associated most clearly with radiating pain below the knee [10], but many studies do not differentiate between back pain and leg pain [11–13].

In the present study including a subset of the patients mentioned above [9], both back pain and leg pain intensity were recorded. All patients had LBP with or without radiating pain [14]. Therefore, a comparison between participants with and without pain was not possibly. Instead, we aimed at analyzing the associations between the pain intensity and MRI findings comparing normal anatomy with degenerated anatomy.

Accordingly, the aims were

1. to analyse for associations between the amount of degenerative manifestations on MRI and the intensity of back + leg pain as well as back pain and leg pain, separately.
2. to clarify which of the three methods of grouping the degenerative manifestations would explain most of the variation in the pain-associations: present vs. absent, sum scores or rank-ordered scores.
3. to establish multivariate models for back + leg pain, back pain and leg pain based on degenerative associations and associations with other pain variables.

## Methods

### Design

Cross-sectional clinical study using standardised blinded MRI description.

### Patients

The patients in the present study belonged to a cohort of patients participating in a clinical intervention study previously reported [14]. They were selected by a one-year study period, in which MRI was performed consecutively. One patient was excluded, as he stayed away from the MRI examination.

Inclusion criteria for joining the clinical intervention study: Partly or fully sick-listed from work for 4–12 weeks due to LBP with or without radiculopathy, LBP should be the prime reason for sick-listing and at least as bothersome as any pain elsewhere, age16–60 years, referred from a well-defined area counting about 280,000 inhabitants, and the patient should be able to speak and understand Danish.

Exclusion criteria: Registered as unemployed, living outside the referral area, continuing or progressive radiculopathy resulting in plans for surgery, low back surgery within the past year, previous lumbar fusion operation, suspected cauda equina syndrome, progressive paresis or other serious back disease (e.g. tumour), pregnancy, known dependency on drugs, or alcohol or primary psychiatric disease.

Patients with spondylolisthesis, severe scoliosis, inflammatory back pain or cancer were excluded [6].

At their first visit, the patients completed a comprehensive questionnaire. Afterwards, a rehabilitation doctor (OKJ) recorded their symptoms and performed a low back examination including measurement of forward flexion, side-flexion and tender point examination. Based on symptoms and physical examination, the patients were classified as having non-specific LBP or radiculopathy. Radiculopathy was defined as structural changes on MRI corresponding to a minimum of one of the following signs: positive Lasegue of 60° or less, missing or inhibited reflex, altered sensation in a dermatome or paresis. In cases of spinal stenosis, spinal claudicatio in combination with MRI findings were sufficient [14, 15].

MRI of the lumbar spine including T1- and T2-weighted sequences was performed within 4–6 weeks at the local hospital using a 0.7-T machine. A few MRI’s were performed with similar techniques at hospitals nearby.

All images were primarily evaluated for clinical use by the rehabilitation doctor, who had access to all clinical data, and the images were described by local specialists in radiology at the hospital as well. A clinical suspicion of radiculopathy was revised, if MRI did not confirm nerve root compromise due to disc herniation or stenosis. Afterwards, all MRI images were transformed to compact discs and blinded, except for identification number, and sent to a specialist in radiology, who was blinded for the clinical data. The MRI images were evaluated and described in accordance with a previously validated protocol [16].

### Questionnaire and clinical data

At the top of the questionnaire, a figure showed the LBP area between the 12th ribs and gluteal folds.

*The LBP rating scale*. This scale has been validated previously [17] and comprises a ‘sum score’ based on questions about worst, average, and actual pain during the preceding 2 weeks. Three numeric rating scales (0–10) were added to a back pain score and a leg pain score, respectively. The sum of these two scores (0–60) comprised the back + leg score.

*Disability in daily life activities.* A validated Danish version of the Roland Morris Questionnaire (RMQ) including 23 items [18].

*Widespread pain (from the Danish version of the General Health Questionnaire):* Affirmative answer on two questions covering the preceding 2 weeks: Much bothered by pain or discomfort in 1) neck, shoulders, arms, hands? 2) back, buttocks, legs, knees and feet?

*Use of pain medication:* 5–7 days per week, 1–4 days per week, 0 days.

*Radiculopathy:* Nerve root pain and at least one of the following clinical signs corresponding with MRI findings: ‘Positive Lasegue ≤ 60°’, ‘missing or inhibited reflex’, ‘altered sensation’ or ‘paresis’.

*Tender point (TP) examination* [7] is a standardized method for assessing diffuse hyperalgesia as in fibromyalgia. A gradually increasing pressure by 1 kg per sec. up to 4 kg was applied by the thumb at 18 spots on the body, symmetrically located on the neck, shoulders, forearms, second ribs, buttocks and legs. Only painful points were counted as positive. The examination technique has been shown to be reliable with good agreement, but less precise [19].

### Ethical approval

Presented in original study [14]. All patients signed informed consent.

### Data analyses

Differences in proportions were analysed by Chi^2^-test, and differences in discrete distributions were analysed by unpaired *t*-test if the distributions were normally distributed and by Wilcoxon rank-sum test if not normally distributed. Spearman’s test was used to correlate not normally distributed variables. Logistic regression was used for analyzing dichotomous outcomes when adjustment was required.

All structural findings on MRI were established as variables in three ways: ‘a max score’, a ‘sum score’ and ‘present vs. absent’. ‘Max scores’ were calculated by rank-ordering the degenerative manifestations and summing up all patients who had the structural finding in question as the most extreme finding (the ‘max’ command in STATA). ‘Sum scores’ were calculated by adding the structural findings on all lumbar levels. ‘Present vs. absent’ were dichotomous variables. Modic changes were analyzed by ‘types’ and ‘sum score’ of volume. The resulting variables were analysed by descriptive statistics.

Back + leg pain, back pain and leg pain were normally distributed and were used as dependent variables in univariate linear regression analyses with the degenerative manifestations and other baseline variables as independent variables. All analyses were adjusted for age and sex. The MRI variables that potentially could cause nerve root compromise were subdivided by radiculopathy, and other variables showing interaction with radiculopathy were also subdivided. Wald’s test was used to test statistical significance for categorical variables. The conditions for using linear regression were checked by normality plots of residuals, residuals versus predicted, and check of leverage and standardised residuals.

Subsequently, multivariate analyses were performed by first analysing degenerative manifestations with back + leg pain, back pain and leg pain as dependent variables to establish models including more than one variable if possible. Afterwards, other explanatory baseline variables were incorporated to achieve higher adjusted R^2^, i.e. the percentage of the variation explained by the linear regression model.

Variables that could be interpreted as caused by pain were not included. Collinearity was checked by multiple correlation analysis.

All analyses were performed by STATA [20], and a significance level of 5 % was chosen.

## Results

Baseline variables are shown in Table 1. Back + leg pain intensity and disability were similar in men and women, but back pain and leg pain differed a little in regard to sex. Radiculopathy was identified in 43 % of the patients, and roughly half of the patients reported pain for more than 3 months.

**Table 1:** Baseline variables

Dependent variables	
Back + leg pain intensity^a^ , mean (SD) (range)	32.3 (11.9) (8, 60)
Women, mean (SD)	32.4 (12.3)
Men, mean (SD)	32.1 (11.6)
Back pain intensity^a^, mean (SD) (range)	17.7 (6.50) (3, 30)
Women, mean (SD)	18.6 (5.8)
Men, mean (SD)	16.6 (7.1)
Leg pain intensity^a^, mean (SD) (range)	14.4 (8.30) (0, 30)
Women, mean (SD)	13.6 (8.5)
Men, mean (SD)	15.3 (8.0)
Independent variables	
MRI findings (presented in Table 2)	
*Demographic and clinical variables*	
Sex: female/all (% female)	75/141 (53)
Age: mean (SD) (range)	41.6 (10.6) (18–60)
Body Mass Index (BMI): mean (SD) (range)	26.8 (4.55) (18.4–40.2)
N^o^ with radiculopathy/all, n (%)	61/141 (43)
Disability (Roland Morris), mean (SD) (range)	15.7 (3.8) (5–23)
Women, mean (SD)	15.6 (4.0)
Men, mean (SD)	15.8 (3.6)
Duration of pain, n (%)	
≤ 3 mo	73 (53)
3-6 mo	38 (27)
7-12 mo	9 (7)
> 12 mo	18 (13)
Much bothered by widespread pain the preceding 2 wk/all , n (%)	23/141 (16)
Using pain medicine 5–7 days pr. wk/ 0–4 days pr. wk/all	86/50/135
Tender points: median (range)	6 (0–18)

Other baseline variables presented elsewhere [6]

^a^on the examination day and the preceding two weeks

By subtracting leg pain intensity from back pain intensity, the relative balance between back pain and leg pain was calculated: Back pain exceeded leg pain in 62 % of all patients. Leg pain was reported as more intense or as intense as back pain in 66 % and 18 % of the patients with and without radiculopathy, respectively. Thus, there was overlap between leg pain and radiculopathy: By cutoff 0.5, the area under the ROC curve for classifying leg pain correctly as radiculopathy was 76 %, sensitivity was 55 % and specificity was 73 %.

### Univariate analyses of degenerative manifestations

The degenerative manifestations shown to the left in Table 2 were predominantly located caudally, the segmental distribution has been presented elsewhere [6].

**Table 2:** Univariate linear regression analyses, adjusted for age and sex

		Back + leg pain (0–60)			Back pain (0–30)			Leg pain (0–30)		
		(*N* = 134)			(*N* = 135)			(*N* = 137)		
*MRI variable*	n (%)	β	(95 % CI)	*p*	β	(95 % CI)	*p*	β	(95 % CI)	*p*
Nucleus signal change										
Max score				0.352*			0.137*			0.772*
Hyperintense										
with band	18 (13)	ref.			ref.			ref.		
Intermediate	119 (84)	1.21	(−4.96, 7.38)	0.699	−0.05	(−3.28, 3.18)	0.975	0.99	(−3.28, 5.27)	0.646
Hypointense	4 (3)	9.63	(−3.67, 22.9)	0.154	6.39	(−0.58, 13.4)	0.072	3.24	(−5.98, 12.5)	0.488
Sum score (ref. with band)		−0.23	(−1.53, 1.07)	0.724	0.24	(−0.45, 0.93)	0.490	−0.43	(−1.32, 0.45)	0.336
Present ( − )	123 (87)	1.44	(−4.74, 7.62)	0.645	0.12	(−3.14, 3.39)	0.941	1.05	(−3.20, 5.31)	0.625
Osteophytes										
Absent	45 (32)									
Sum score (ref. absent)		0.65	(−0.31, 1.61)	0.184	0.22	(−0.28, 0.73)	0.382	0.38	(−0.26, 1.03)	0.242
Present ( − )	96 (68)	1.55	(−3.15, 6.25)	0.469	−0.22	(−2.69, 2.25)	0.859	2.05	(−1.14, 5.25)	0.207
Disc height reduction										
Max score				0.250*			0.631*			0.115*
Absent	28 (20)	ref.			ref.			ref.		
Slight	40 (28)	5.06	(−0.85, 11.0)	0.093	0.98	(−2.15, 4.11)	0.536	3.97	(−0.06, 8.02)	0.054
Moderate	63 (45)	1.89	(−3.73, 7.52)	0.507	−0.77	(−3.76, 2.32)	0.614	2.18	(−1.65, 6.00)	0.263
Severe	10 (7)	6.81	(−2.52, 16.1)	0.151	0.15	(−4.82, 5.13)	0.951	6.64	(0.25, 13.0)	*0.042*
Sum score (ref. absent)		0.36	(−0.57, 1.29)	0.447	0.08	(−0.42, 0.57)	0.757	0.24	(−0.39, 0.88)	0.453
Present ( − )	113 (80)	3.53	(−1.63, 8.70)	0.178	0.02	(−2.72, 2.76)	0.987	3.21	(−0.33, 6.74)	0.075
High Intensity Zone										
Absent	42 (30)									
Sum score (ref. absent)										
Present ( − )	99 (70)	−0.05	(−2.40, 2.30)	0.969	−0.41	(−1.65, 0.83)	0.513	0.41	(−1.20, 2.00)	0.617
		2.74	(−1.83, 7.31)	0.238	−0.66	(−3.07, 1.76)	0.593	3.53	(0.46, 6.60)	*0.025*
Protrusion/ herniation										
Max score				0.548*			0.154*			0.032*
Absent	15 (11)	ref.			ref.			ref.		
Bulging	23 (16)	−1.94	(−10.3, 6.31)	0.637	0.12	(−4.19, 4.43)	0.956	−2.55	(−8.03, 2.94)	0.360
Protrusion										
*Broad*	7 (5)	−2.55	(−13.5, 8.42)	0.646	0.09	(−5.61, 5.79)	0.976	−2.63	(−9.51, 4.70)	0.479
*Focal*	48 (34)	2.77	(−4.49, 10.0)	0.452	1.48	(−2.29, 5.25)	0.439	1.29	(−3.56, 6.13)	0.600
Extrusion	40 (28)	1.28	(−6.37, 8.94)	0.740	−2.41	(−6.37, 1.54)	0.229	3.21	(−1.87, 8.29)	0.214
Sequestration	8 (6)	6.18	(−5.35, 17.7)	0.291	−1.49	(−7.48, 4.50)	0.624	7.65	(−0.05, 15.3)	0.051
Sum score (ref. absent)		0.03	(−0.82, 0.86)	0.939	−0.24	(−0.69, 0.21)	0.290	0.29	(−0.29, 0.86)	0.328
Present (−)	126 (89)	1.78	(−5.61, 7.97)	0.732	−0.05	(−3.63, 3.54)	0.979	0.97	(−3.70, 5.65)	0.681
MRI nerve root sign^a^										
Max score				0.688*			0.023*			0.104*
No touch	52 (37)	ref.			ref.			ref.		
Touch	31 (22)	2.95	(−2.59, 8.49)	0.294	1.79	(−1.05, 4.62)	0.214	1.42	(−2.31, 5.16)	0.452
Displacement	37 (26)	2.59	(−2.87, 8.05)	0.349	−0.98	(−3.77, 1.82)	0.490	3.45	(−0.19, 7.08)	0.063
Compression	21 (15)	1.02	(−5.80, 7.84)	0.769	−3.90	(−7.30, −0.49)	*0.025*	5.09	(0.55, 9.62)	*0.028*
Sum score (ref. no touch)		0.20	(−1.46, 1.86)	0.813	−0.82	(−1.67, 0.04)	0.062	0.90	(−0.20, 2.00)	0.109
Present (−)	89 (73)	2.43	(−1.97, 6.83)	0.276	−0.49	(−2.81, 1.84)	0.679	2.97	(0.00, 5.94)	0.050
Spinal stenosis										
Max score				0.587*			0.426*			0.894*
Absent	123 (87)	ref.								
Relative	14 (10)	3.81	(−3.48, 11.1)	0.303	2.44	(−1.41, 6.28)	0.212	0.51	(−4.35, 5.38)	0.768
Severe	4 (3)	1.11	(−11.4, 13.6)	0.861	−0.74	(−7.34, 5.86)	0.825	1.95	(−6.70, 10.6)	0.657
Sum score (ref. absent)		1.24	(−1.63, 4.11)	0.393	0.45	(−1.06, 1.97)	0.554	0.52	(−1.41, 2.45)	0.594
Present (−)	18 (13)	1.90	(−3.12, 6.92)	0.455	0.79	(−1.86, 3.44)	0.556	0.78	(−2.63, 4.18)	0.653
Modic changes										
Type				0.242*			0.109*			0.708*
Absent	57 (40)	ref.			ref.			ref.		
Type 1	25 (18)	0.72	(−5.29, 6.73)	0.813	1.52	(−1.63, 4.66)	0.341	−0.96	(−5.08, 3.16)	0.609
Type 2	59 (42)	−3.54	(−8.47, 1.39)	0.157	−1.73	(−4.30, 0.85)	0.187	−1.42	(−4.83, 1.99)	0.412
Sum score (ref. abs.)		0.13	(−0.51, 0.77)	0.689	0.08	(−0.26, 0.42)	0.642	0.05	(−0.39, 0.49)	0.812
Present (ref. abs.)	84 (60)	−2.12	(−6.65, 2.41)	0.356	−0.64	(−3.03, 1.74)	0.594	−1.26	(−4.37, 1.84)	0.464

*β* regression coefficient; *CI* confidence interval; *ref* reference. *P*-values less than 0.05 indicated by *Italics*

Back + leg pain, back pain and leg pain were dependent variables. The degenerative manifestations were described blinded and standardised and expressed as ‘max scores’, ‘sum scores’ and ‘present vs. not present’. A ‘max score’ shows the number of patients with the manifestation indicated as the worst manifestation. The distribution of degenerative manifestations across the lumbar segments has been published elsewhere [6]

The numbers in subgroups not adjusted for missing values

*Overall *p* by Wald’s test

^a^The highest adj. R^2^ = 0.10 (back pain intensity), all other < 0.10

### Summary of MRI associations in Tables 2 and 3

The relative proportion of patients with clinical radiculopathy increased by increasing degree of disc herniation and MRI nerve root sign (‘max score’ left column, Table 3)

**Table 3:** Univariate linear regression analyses as measured by max scores, subdivided by radiculopathy

		Back + leg pain (*N* = 134)			Back pain (*N* = 135)			Leg pain (*N* = 137)		
*MRI variable*	n (%)	β	(95 % CI)	*p*	β	(95 % CI)	*p*	β	(95 % CI)	*p*
Osteophytes^a^				0.003*			0.810*			<0.001*
Absent	45 (32)	ref.			ref.			ref.		
Present										
–radiculopathy	48 (34)	−2.52	(−7.64, 2.59)	0.331	0.19	(−2.60, 2.99)	0.892	−2.52	(−5.74, 0.71)	0.125
+radiculopathy	48 (34)	6.00	(0.78, 11.2)	*0.025*	−0.68	(−3.54, 2.19)	0.641	7.00	(3.72, 10.3)	*<0.001*
High intensity zone				0.034*			0.528*			<0.001*
Absent	42 (30)	ref.			ref.			ref.		
Present										
–radiculopathy	43 (31)	−0.33	(−5.52, 4.48)	0.899	0.05	(−2.74, 2.85)	0.970	0.34	(−3.64, 2.97)	0.841
+radiculopathy	56 (40)	5.63	(0.52, 10.7)	*0.031*	−1.30	(−4.04, 1.44)	0.348	7.15	(3.90, 10.4)	*<0.001*
Protrusion/herniat^a^.				0.116*			0.228*			<0.001*
Absent	15 (11)	ref.			ref.			ref.		
Bulging										
–radiculopathy	18 (13)	−4.76	(−13.2, 3.71)	0.268	−0.71	(−5.23, 3.81)	0.755	−4.43	(−9.74, 0.89)	0.102
+radiculopathy	5 (4)	9.26	(−3.16, 21.6)	0.143	3.67	(−2.95, 10.3)	0.275	5.67	(−2.23, 13.6)	0.158
Protrusion										
*Broad*										
–radiculopathy	6 (4)	−4.31	(−15.6, 6.95)	0.450	−0.71	(−6.71, 5.29)	0.815	−3.59	(−10.8, 3.57)	0.323
+radiculopathy	1 (1)	9.37	(−15.2, 33.9)	0.451	5.32	(−7.76, 18.4)	0.422	4.14	(−11.5, 19.7)	0.601
*Focal*										
–radiculopathy	29 (21)	0.43	(−7.15, 8.01)	0.910	2.20	(−1.84, 6.24)	0.283	−1.74	(−6.56, 3.08)	0.477
+radiculopathy	19 (13)	7.40	(−0.95, 15.8)	0.082	0.55	(−3.90, 5.00)	0.806	6.87	(1.56, 12.2)	*0.012*
Extrusion										
–radiculopathy	12 (9)	0.62	(−8.82, 10.1)	0.897	−1.29	(−6.33, 3.74)	0.613	0.78	(−5.09, 6.64)	0.793
+radiculopathy	28 (20)	2.51	(−5.45, 10.5)	0.533	−2.77	(−6.97, 1.43)	0.193	5.21	(0.16, 10.2)	*0.043*
Sequestration										
–radiculopathy	0	–	–	–	–	–	–	–	–	–
+radiculopathy	8 (6)	7.39	(−3.91, 18.7)	0.198	−1.47	(−7.49, 4.55)	0.630	8.89	(1.71, 16.1)	*0.016*
MRI nerve root sign				0.033*			0.056*			<0.001*
No touch	52 (37)	ref.			ref.			ref.		
Touch										
–radiculopathy	19 (13)	−2.62	(−8.84, 3.60)	0.406	0.78	(−2.52, 4.09)	0.639	−3.17	(−7.19, 0.86)	0.122
+radiculopathy	12 (9)	13.0	(5.18, 20.8)	*0.001*	3.52	(−0.62, 7.66)	0.095	9.73	(4.68, 14.8)	*<0.001*
Displacement										
–radiculopathy	15 (11)	1.18	(−6.02, 8.39)	0.745	−0.09	(−3.92, 3.73)	0.961	0.62	(−3.92, 5.15)	0.789
+radiculopathy	22 (16)	4.08	(−2.10, 10.3)	0.194	−1.51	(−4.79, 1.78)	0.365	5.90	(1.91, 9.90)	*0.004*
Compression										
–radiculopathy	3 (2)	−1.50	(−15.3, 12.3)	0.831	−0.61	(−7.96, 6.75)	0.871	0.60	(−9.58, 8.38)	0.895
+radiculopathy	18 (13)	2.18	(−4.92, 9.29)	0.544	−4.53	(−8.18, −0.87)	*0.016*	6.86	(2.36, 11.4)	*0.003*
Spinal stenosis ^b^				0.198*			0.532*			0.084*
Absent	123 (87)	ref.			ref.			ref.		
Present										
–radiculopathy	7 (5)	−2.77	(−12.9, 7.37)	0.590	0.56	(−4.83, 5.95)	0.837	−4.54	(−11.0, 1.93)	0.167
+radiculopathy	11 (8)	6.62	(−1.29, 15.5)	0.100	2.39	(−1.82, 6.60)	0.263	4.39	(−1.01, 9.79)	0.110

β regression coefficient; *CI* confidence interval; *ref* reference. *P*-values less than 0.05 indicated by *Italics*

Back + leg pain, back pain and leg pain intensity were dependent variables, all analyses adjusted for age and sex. The numbers in subgroups not adjusted for missing values

The estimates for small subgroups (<5) should not be considered reliable

*Overall p by Wald’s test

^a^The highest adj. R^2^ = 0.22 (leg pain), the second highest disc herniation (leg pain): 0.19

^b^‘Relative and severe’ collapsed because of small number of ‘severe’

.

Osteophytes and high intensity zone (HIZ) were not associated with back pain and were only associated with leg pain, when radiculopathy was present (Tables 2 and 3).

**Fig. 1:**
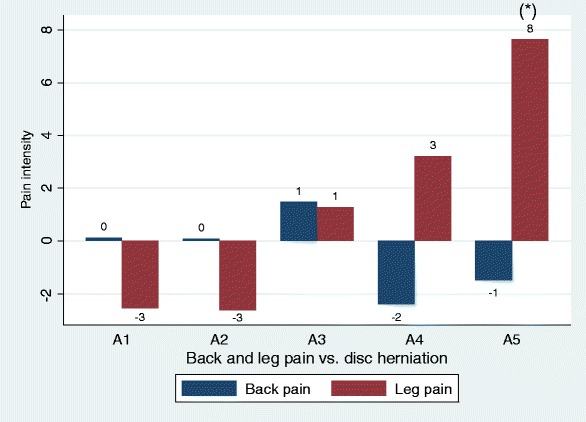
Back pain and leg pain in relation to different types of disc herniations. Overall, only associations with leg pain were statistically significant (Table 2), statistical significance by type indicated by ‘*’. Negative values should be interpreted as less pain as compared to the reference group (no disc herniation). A1, bulging; A2, broad protrusion; A3, focal protrusion; A4, extrusion; A5, sequester

**Fig. 2:**
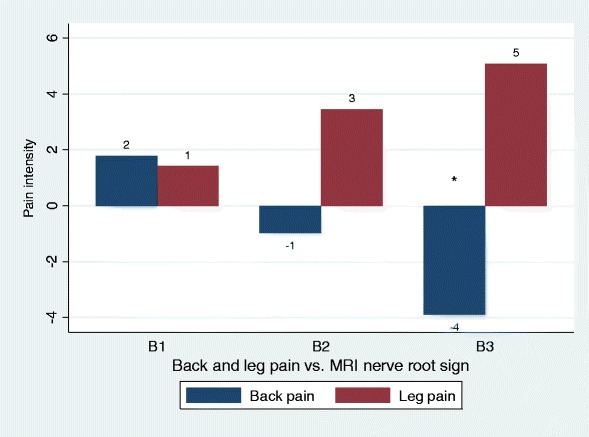
Back pain and leg pain in relation to different types of MRI nerve root sign. Overall, only associations with back pain were statistically significant (Table 2), statistical significance by type indicated by ‘*’. Negative values should be interpreted as less pain as compared to the reference group (no MRI nerve root sign). B1, nerve root touch; B2, nerve root displacement; B3, nerve root compression

**Fig. 3:**
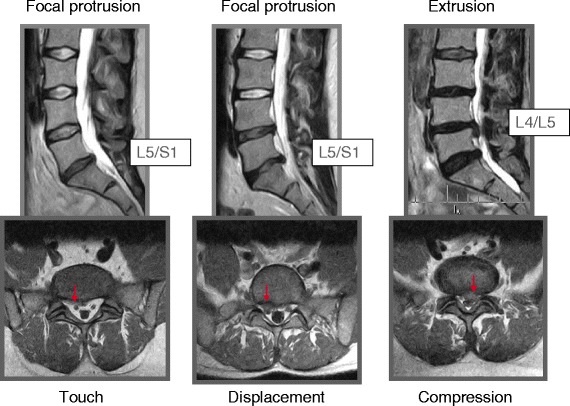
Focal protrusion with nerve root touch (*left*) or displacement (*middle*) and extrusion with nerve root compression (*right*)

Disc herniations were not associated with back pain, but MRI nerve root compression was associated with less back pain as compared to the reference group (Table 2 Figs. 1 and 2). Nerve root touch (Fig. 3) tended to be positively associated with back pain in contrast to nerve root displacement or compression (Fig. 2 and 3).

**Fig. 4:**
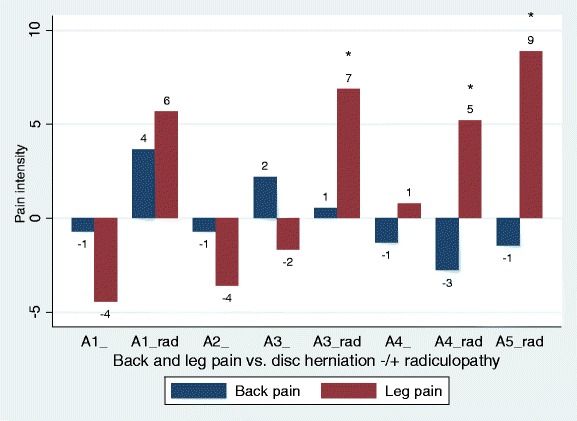
Back pain and leg pain in relation to different types of disc herniations with and without radiculopathy. Overall, only associations with leg pain were statistically significant (Table 3), statistical significance by type indicated by ‘*’. Negative values should be interpreted as less pain as compared to the reference group (no disc herniation). A1, bulging; A2, broad protrusion; A3, focal protrusion; A4, extrusion; A5, sequester, ‘rad’ indicating + radiculopathy. A2_rad and A5_ omitted as these subgroups included 1 and 0 patients, respectively

**Fig. 5:**
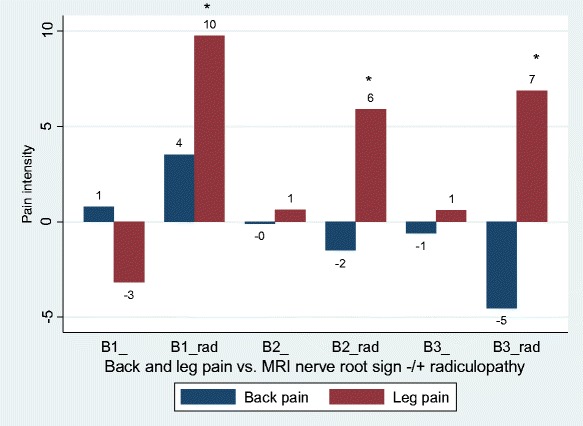
Back pain and leg pain in relation to different types of MRI nerve root sign with and without radiculopathy. Overall, only associations with leg pain were statistically significant, back pain being borderline (*p* = 0.056, Table 3). Statistical significance by type of MRI nerve root sign indicated by ‘*’. Negative values should be interpreted as less pain as compared to the reference group (no MRI nerve root sign). B1, nerve root touch; B2, nerve root displacement; B3, nerve root compression, ‘rad’ indicating + radiculopathy

Disc herniations and MRI nerve root signs were only associated with leg pain when radiculopathy was present (Tables 2 and 3 Figs. 4 and 5). Nerve root touch was as much associated with leg pain as nerve root displacement and compression in patients with radiculopathy (Table 3, Fig. 5).

There were no statistically significant associations for nucleus signal changes, disc height reduction, spinal stenosis or Modic changes (Tables 2 and 3).

Generally, the ‘max scores’ showed the differences more clearly than the ‘sum scores’ and ‘present vs. absent’ analysis. When analyzing the ‘max score’ associations subdivided by radiculopathy, more statistically significant associations appeared (Table 3), and adjusted R^2^ increased owing to the strong association between radiculopathy and leg pain as shown in Table 4.

**Table 4:** Univariate linear regression analyses of demographic and clinical variables

	Back + leg pain (0–60) (*N* = 134)			Back pain (0–30)			Leg pain (0–30)		
				(*N* = 135)			(*N* = 137)		
*Baseline demographic and clinical variables*	β	(95 % CI)	*p*	β	(95 % CI)	*p*	β	(95 % CI)	*p*
Sex , ref. female	−0.25	(−4.35, 3.86)	0.906	−2.01	(−4.21, 0.19)	0.073	1.72	(−1.08, 4.53)	0.226
Age, pr. y, ref. 18 y	−0.06	(−0.25, 0.14)	0.576	−1.35	(−0.24, −0.03)	*0.011*	0.08	(−0.06, 0.21)	0.252
BMI, pr. unit, ref. 18.4	0.17	(−0.28, 0.62)	0.449	0.22	(−0.01, 0.46)	0.062	−0.01	(−0.32, 0.30)	0.947
Radiculopathy, ref. no^a^	6.52	(2.20, 10.8)	*0.003*	−1.21	(−3.54, 1.11)	0.302	7.95	(5.22, 10.7)	*<0.001*
Disability, pr. unit, ref. 5^a^	1.50	(1.02, 1.98)	*<0.001*	0.57	(0.30, 0.84)	*<0.001*	0.93	(0.59, 1.27)	<0.*001*
Duration of pain			0.080*			0.029*			0.083*
≤ 3 mo	ref.			ref.			ref.		
3-6 mo	2.30	(−2.51, 7.10)	0.346	2.55	(0.07, 5.02)	*0.044*	0.18	(−3.12, 3.48)	0.914
7-12 mo	10.9	(2.38, 19.3)	*0.012*	5.24	(0.86, 9.62)	*0.019*	6.06	(0.21, 11.9)	*0.043*
> 12 mo	0.19	(−6.32, 6.70)	0.954	3.32	(−0.03, 6.68)	0.052	−2.69	(−7.16, 1.79)	0.238
Widespread pain yes, ref. no	9.46	(3.68, 15.2)	*0.002*	3.02	(−0.11 , 6.14)	0.058	6.69	(2.71 , 10.7)	*0.001*
Use of pain medicine 5–7 days pr. wk, ref. 0–4 days	6.34	(2.08, 10.6)	*0.004*	2.45	(0.19 , 4.72)	*0.034*	3.45	(0.52 , 6.39)	*0.021*
Tender points, pr. point, ref. 0	0.15	(−0.39, 0.69)	0.579	0.35	(0.08, 0.63)	*0.012*	−0.21	(−0.59, 0.15)	0.247

β regression coefficient; *CI* confidence interval; *ref* reference. *P*-values less than 0.05 indicated by *Italics*

Back + leg pain, back pain and leg pain intensity were dependent variables. All analyses adjusted for age and sex, except age and sex

*Overall *p* by Wald’s test

^a^The highest adj. R^2^ for dichotomous variables was 0.20 (radiculopathy – leg pain) and for variables with discrete distributions 0.21 (disability – leg pain)

When adjusting for leg pain by subtracting leg pain from back pain, nerve root touch also was positively associated with back pain in patients without radiculopathy (β = 4.19 (0.27–8.10), *p* = 0.036, data not shown in the Tables).

### Univariate analyses of demographic and clinical variables

Back pain was negatively associated with age, and leg pain tended to be positively associated with age (Table 4). BMI was not statistically significantly associated with any of the outcomes (Table 4).

Radiculopathy was strongly associated with leg pain, not with back pain. Disability and use of pain medicine were associated with all three outcomes. Back pain was higher in patients with pain duration >3 months, leg pain not statistically significant (Table 4).

Widespread pain was associated with leg pain, not with back pain (Table 4). The same was true when only including patients with non-specific LBP (back pain β = 3.3, *p* = 0.054; leg pain β = 10.7, *p* < 0.001).

**Fig. 6:**
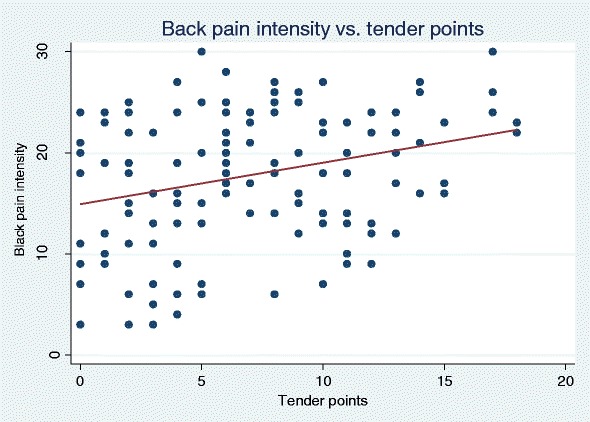
The association between back pain intensity and tender points. The slope β was 0.41 when unadjusted for age and sex. The broad cloud of dots reflects the considerable variability, though statistically significant as shown in Table 4 and 5

TPs were associated with back pain (Table 4, Fig. 6), but not with leg pain. When only considering patients with non-specific LBP, β turned positive for leg pain (back pain β = 0.44, *p* = 0.010; leg pain β = 0.35, *p* = 0.151).

TPs correlated with widespread pain (Wilcoxon’s ranksum test, *p* = 0.0002), but there was only partly overlap. More than 10 TPs were present in 31 patients (22 %), and widespread pain was reported by 23 patients (16 %). A combination of more than 10 TPs and widespread pain was only present in 11 patients (8 %).

### Other associations

Except for HIZ displaying only borderline association with age (*p* < 0.1), all other degenerative manifestations were associated with age (p varying form 0.02 to 0.0001). Disc herniation, MRI nerve root sign and spinal stenosis were more prevalent in men than in women (p varying from 0.03 to 0.001), but there was no statistically significant sex difference in regard to other degenerative manifestations. More men than women had radiculopathy (66 %, *p* < 0.001).

HIZ was associated with disc herniation (*p* < 0.001), and 76 of 99 HIZ were present in patients with focal protrusions or extrusions. All degenerative manifestations were correlated, especially disc herniation and MRI nerve root sign (Spearman’s rho 0.79, *p* < 0.001). Nerve root touch was most often seen in relation to focal protrusion (21 of 31 nerve root touch cases).

### Multivariate analyses also including other pain variables

Back + leg pain and leg pain associations were similar, except for MRI nerve root sign explaining more of the pain in the former and disc herniation in the latter (Table 5). Widespread pain contributed further to the two models explained leg pain in a subset of patients without radiculopathy. Only 14 % of the back + leg pain variation was explained, whereas 31 % of the leg pain variation was explained. Age was only statistically significant in the leg pain model. MRI findings explained most of the variation.

**Table 5:**
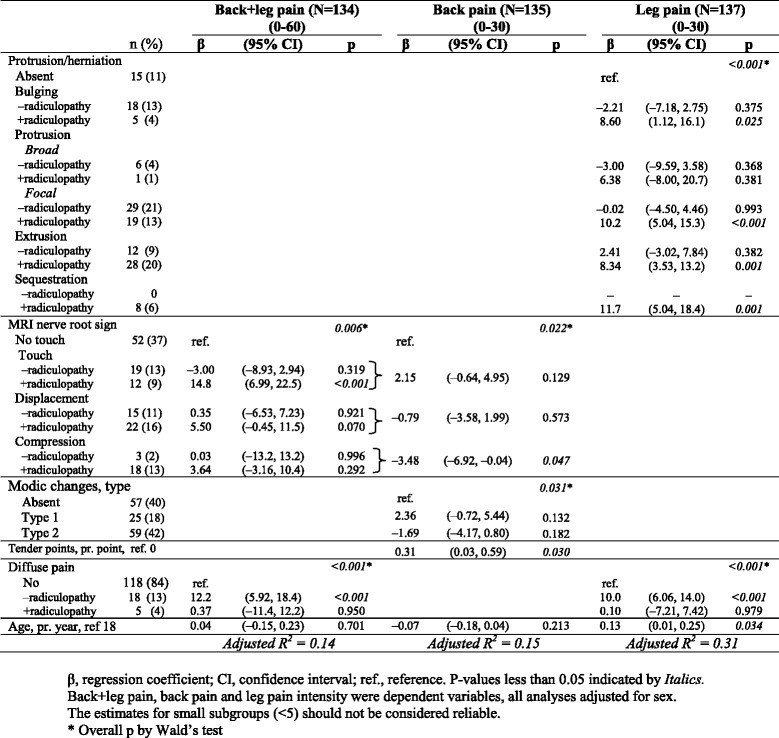
Three multivariate models including MRI variables and other explanatory variables

Back pain was associated with type 1 Modic changes as compared to type 2 Modic changes when adjusted for MRI nerve root sign (Table 5). Other combinations of MRI variables were not possible due to collinearity. MRI nerve root displacement and compression were associated with less back pain, and nerve root touch tended to be associated with more back pain. Only the tender point variable contributed to this model. Thus, 15 % of the back pain variation was explained, and MRI findings explained most of the variation.

Disability and use of pain medicine were not included in the models, as they were considered caused by the pain, not explaining the pain. Sex and BMI did not contribute to the models, and pain duration was not included in the back pain model, as it explained less of the back pain than the MRI associations.

## Discussion

In the present study, 57 % and 43 % of the patients were classified clinically as non-specific LBP and radiculopathy, respectively, Sixty-two percent of the patients reported more back pain than leg pain.

Back pain tended to be dominant in women and leg pain dominant in men. However, back + leg pain and disability were strikingly similar in the two sexes probably owing to common selection criteria in terms of 1–4 months of sick-listing. There was a preponderance of men among the patients with radiculopathy, which is in agreement with other studies [21, 22]. However, the literature is not consistent in regard to sex-difference of degenerative manifestations: X-ray studies have shown osteophytes and end plate sclerosis more prevalent in men, but disc height reductions more prevalent in women [23, 24]. Furthermore, gender was not associated with the one-year prognosis in the present study [15, 25] or in LBP studies in general [26].

In the multivariate analyses, age was positively associated with leg pain probably reflecting the age-association with degenerative manifestations also demonstrated in other studies [27].

Accordingly, all analyses were adjusted for age and sex.

The associations between pain and MRI findings were best demonstrated by the ‘max score’ analyses. However, only 15 % of the back pain was explained by MRI nerve root sign, type 1 Modic changes and tender points. In contrast, the leg pain was better explained by age, degenerative manifestations and widespread pain accounting for 31 % of the variation.

Finally, back + leg pain was least explained reflecting different associations for back pain and leg pain. Nonetheless, we still consider the back + leg pain variable important, as it was previously identified as one of only two variables contributing in explaining all three outcomes at 1 year: pain, disability and return to work [15, 25]. The other variable was type 1 Modic changes [6].

The lack of association between back pain and disc herniation was in good accordance with some previous studies [1, 28]. It is also illustrated by the high prevalence of asymptomatic disc herniations [4]. However, most studies have shown association between disc herniation and LBP the past year [4, 29]. A cohort study with follow-up imaging after 3 years showed associations between previous pain and the presence of disc extrusion, central stenosis and nerve root compromise, however, not other degenerative manifestations [30]. Thus, disc herniation may be associated with previous pain rather than actual pain.

Back pain was associated with nerve root touch especially in patients with radiculopathy, but not nerve root displacement or compression. Thus, it may be hypothesized that nerve root touch may be more important for back pain than just the presence of disc herniation.

Type 1 Modic changes are characterized by more inflammatory changes than type 2 changes [31] which may explain the association with back pain. The association demonstrated here is in accordance with the literature [32, 33]. Although in the present study associated with back pain and not leg pain, it may not be concluded that type 1 Modic changes do not cause leg pain.

Although disc herniations frequently occur without pain [4, 34], leg pain is often well explained by disc herniations, especially extrusions and sequesters, which was demonstrated here as elsewhere [10, 35]. However, we found no association in patients without radiculopathy, which may be due to the small sample size. But it also may reflect that disc herniation seldom causes leg pain in the absence of radiculopathy.

As expected, nerve root touch, displacement and compression were also closely associated with leg pain, but clinical radiculopathy was only present in about two thirds of these (43 % vs. 63 %). The explanation may be chemical. Radiculopathy is dependent on both pressure and chemical substances predominantly induced by contact with nucleus pulposus tissue [36]. We found nerve root touch as much associated with leg pain as nerve root displacement or compression and also with back pain. This also may indicate that the chemical component is more important than the mechanical component, which was supported by another study [28]. That study showed no significant differences in regard to degenerative manifestations between a group with radiculopathy and a matched control group, except for nerve root impingement. Some studies have shown effect of tumour necrosis factor inhibitors or anti-interleukin 6 in patients with radiculopathy. [36–38]. In one study in patients with sciatica, a TNF-alfa inhibitor (eternacept) infiltrated on the nerve root was more effective than dexamethasone at 1 month [37], in another study in patients with lumbar stenosis, anti-interleukin 6 infiltrated on the nerve root was more effective than dexamethasone at 1 month [38]. Thus in the future, we may learn to manage the chemical component more specifically by biologic therapy in some of the patients with radiculopathy.

HIZ is often believed to be responsible for back pain, although not confirmed by two population studies [1, 39], one of these including more than 1000 patients [39]. In the present study, HIZ was only associated with leg pain in patients with radiculopathy, which partly may depend on its association with disc herniation.

Similarly, osteophytes were only associated with leg pain in patients with radiculopathy. This may be explained by the osteophytes’ potential of being space occupying. We have not been able to find other MRI studies highlighting this aspect, but population studies using X-rays have shown just modest associations with LBP [40].

Associations between degenerative manifestations and back pain may have been overshadowed by other pain mechanisms as for instance descending anti-nociceptive mechanisms: The TP variable contributed to the multivariate back pain model, and patients with more than 10 tender points had 4 units higher back pain intensity than patients with less than 2 tender points. Tender points may explain some cases of back pain by a fibromyalgia-like pain mechanism, as tender points were previously shown to be positively associated with back pain and negatively associated with radiculopathy and disc height reduction [9]. Of course tender points cannot be regarded as a very precise measure, but rather may be understood as some sort of standardised measure of diffuse tenderness.

Widespread pain was associated with both tender points and leg pain, but not with back pain. It did explain leg pain in some of the patients without radiculopathy. Widespread pain is reported by 10–13 % in the general population [41] and was present in 16 % of the patients. Tender points and widespread pain may indicate sensitization of the nociceptive system, but notably, they played different roles in relation to back pain and leg pain in the present study.

Tender points were previously shown to be associated with one-year pain and disability in the multivariate analyses, whereas widespread pain was only associated with the outcome in the univariate analyses [15].

### Strengths

The statistical analyses were performed in three different ways allowing us to compare the result of ‘max scores’ ‘sum scores’ and ‘present vs. absent’.

The present pain registration provided a better mapping of back pain and leg pain than in most other studies. The classification in non-specific LBP or radiculopathy took place before and independent of the standardised MRI description that has been validated previously [16].

The present population included patients referred by general practitioners for secondary health care [14], but all patients were selected by sick-listing criteria and were probably not subjected to other sorts of bias such as economic aspects, care seeking behaviour or expectations or plans for surgery.

Activities of daily living were importantly affected, as the mean Roland Morris score was 16, a high level in comparison with other LPB populations [42].

#### Limitations

The present study was cross-sectional and therefore cannot prove causal relationships. The total number of patients was limited and defined by pragmatic reasons and not by power calculation. This resulted in small subgroups implying a risk for overlooking associations with MRI findings, and it may also explain the lack of associations with spinal stenosis. Some structural findings were not included such as disorders like Scheuermann or spondylolisthesis. Widespread pain recording only covered the preceding 2 weeks. Multiple testing implied a risk for coincidental findings.

#### Perspectives

The lack of associations between back pain and all degenerative manifestations except type 1 Modic changes highlights the need for caution, when degenerative manifestations on MRI are interpreted and explained to patients with non-specific LBP. Usually, we do not know the exact cause of pain in patients with non-specific LBP [43], and this is still so even when MRI of the lumbar spine is available.

There is a need for more research in immunological processes and chemical substances in the spinal canal influencing pain processing.

## Conclusions

The associations between MRI findings and pain were best demonstrated by the ‘max score’ analyses, i.e. by subclassifying the patients in relation to the most severely degenerated segment.

The present study confirmed the importance of identifying patients with radiculopathy, not only for selection purpose for surgery, but also in order to better understand pain.

Degenerative MRI findings explained disappointingly little of the back pain, but explained more of the leg pain, especially after incorporating the clinical classification of radiculopathy. Nerve root touch was both associated with back pain and leg pain, but nerve root displacement or compression was only associated with leg pain. Disc herniation, HIZ or osteophytes were not associated with back pain, but with leg pain in patients with radiculopathy. Diffuse hyperalgesia, as measured by tender points, contributed in explaining the back pain. Widespread pain contributed in explaining the leg pain in patients without radiculopathy.
